# Deservingness and temporal borders: the reproduction of global mobility hierarchies in Swedish family reunification

**DOI:** 10.3389/fsoc.2024.1427262

**Published:** 2024-11-05

**Authors:** Hilda Gustafsson, Rikard Engblom

**Affiliations:** ^1^Department of Global Political Studies, Malmö Institute for Migration Studies, Malmö University, Malmö, Sweden; ^2^The School of Behavioural, Social and Legal Sciences, Örebro University, Örebro, Sweden; ^3^Department of Cultural Anthropology and Ethnology, Uppsala University, Uppsala, Sweden

**Keywords:** temporal borders, deservingness, mobility regime, family reunification, migration, inequality, waiting

## Abstract

European immigration policy is increasingly selective and stratified, favoring immigrants considered productive in the eyes of society. Using the case of Swedish family reunification, this article investigates how ideas of deservingness underlie this selection process and how it intersects with temporal bordering, impacting hierarchies of transnational mobility. Through qualitative interviews with individuals across a spectrum of legal statuses, the study finds that the increased connection between immigration policy and the housing and labor markets, combined with restrictions concerning visas, age, and legal status, induce and reproduce inequalities in waiting times and access to reunification. Within these restrictions, however, families find ways to circumvent the wait and get family time. The study contributes to the temporal turn in migration studies by exploring reunification among families with diverse backgrounds, complementing previous literature’s focus on the experiences of forced migrants. By considering how deservingness and temporal bordering shape mobility, the article offers both conceptual and empirical contributions to mobility and migration studies. Ultimately, the study brings forward a nuanced analysis of the consequences of restrictive shifts in Swedish immigration policy, contributing to the broader understanding of the current, transnational, mobility regimes.

## 1 Introduction

Over the past decades, European states have increasingly controlled immigration through selection and stratification based on immigrant categories such as workers, students, or refugees. This development has resulted in more open policies for some groups, while others, especially forced migrants, face great obstacles and very few routes to settlement in safety (De Haas et al., 2018; UNHCR, 2024). This shift has directed scholars’ attention to the restrictive dynamics of global mobility regimes and the hierarchization of transnational mobility.

Recent studies show that the current European mobility regime is increasingly based on *deservingness*, requiring immigrants to prove themselves worthy by learning the language, following laws and customs, and achieving financial self-sufficiency through employment to qualify for residence permits and rights (Bech et al., 2017; Hameršak et al., 2020; Marchetti, 2020; Mezzadra, 2019; Schindel, 2022). In Sweden, for example, refugees receive a time-limited residence permit if granted asylum, and must obtain a stable income to become eligible for permanent residency (SFS, 2005:716). A logic of deservingness discerns supposedly easily integrable individuals from those considered undesirable, reproducing social hierarchies, according to the critique. Moreover, with an intensified relation between labor market integration and immigration policy, power is transferred from state bureaucracies to actors on the labor market, confirming Sandro Mezzadra’s and Brett Neilson’s argument that capital plays a central role in configuring contemporary borders, and shaping mobilities (Mezzadra and Neilson, 2013).

Alongside deservingness, *temporal borders*—i.e. different mechanisms that regulate and control peoples’ time—have become an integral and inescapable part of the mobility regime (Mezzadra and Neilson, 2013). Öberg and Sager (2017) consider the shift towards time-limited residence permits a consolidation of temporariness in Swedish migration policy. Imposing temporariness, they suggest, does not only affect those who are applying for residency within Sweden, but effectively discourages migrants who cannot make themselves productive in the Swedish labor market from arriving (see also Andersson, 2014a).

Stricter requirements for entry increase the amount of people waiting both outside and within the prevailing European mobility regime. The immobility and “stolen time” (Khosravi, 2021; Mulinari, 2024) caused by restrictive immigration measures have been explored in existing literature, arguing that forcing people to wait is one way in which power asymmetries are reproduced. However, the main focus has often been on forced migration (Bendixsen and Eriksen, 2018; Drangsland K. A., 2020; Griffiths, 2017; Khosravi, 2021; Mulinari, 2021; Tazzioli, 2018), with limited attention given to how temporal borders affect other groups.

This article looks beyond forced migration by analyzing qualitative interviews with individuals who applied for reunification of various backgrounds in terms of residence permit type, nationality, level of education, age, financial situation, and abilities. We investigate the dynamics of deservingness and temporal borders in the recent, stringent family reunification landscape of Sweden, and how the two stratify the privilege of mobility and access to reunification.

The article contributes to the so-called *temporal turn* in migration studies (Baas and Yeoh, 2019) by exploring how current reunification regulations and deservingness subject transnational families to prolonged periods of uncertain waiting. It also provides both conceptual and empirical insights to the literature on family reunification by analyzing how deservingness and temporal bordering effectively reinforce a socially stratified and unequal distribution of reunification.

Family migration is an interdisciplinary research field that spans across a number of interlaced themes, including transnational families (Levitt, 2001; Baldassar et al., 2007), family separation (Tiilikainen et al., 2023; Leinonen and Pellander, 2020; Stange and Stark, 2019; Enchautegui and Menjívar, 2015; Suârez-Orozco et al., 2002), mixed-status families (Yoshikawa, 2011), gender roles (Asis et al., 2004; Levitt and Jaworsky, 2007) and the sending of remittances (Yeoh et al., 2013). Family reunification is rarely the main focus in these studies, however. Important exceptions are Bonjour and de Hart (2013) who have explored how restrictions on family reunification policy are influenced by Dutch identity formation and gender norms (de Hart and Besselsen, 2020; Bonjour and de Hart, 2021), as well as Kofman (2018) who found that maintenance requirements for reunification signal that citizens and non-citizens are “deemed not to deserve to benefit from the right to bring their family members into the national community” (p. 42). In a Swedish context, Rosén’s (2010) doctoral thesis on caseworkers’ assessments of relationship seriousness found discrepancies in both case handling and decisions based on sponsors’ (i.e., the family member in Sweden) backgrounds in terms of education, employment status, and citizenship. Bech et al. (2017) and Borevi (2014, 2018) have focused on the previous uniqueness and subsequent shift of Sweden’s reunification policy. The role of temporality in Swedish family reunification literature remains scarce, yet some recent exceptions include Gustafsson’s (2022) article on collective aspects of waiting among transnational families, Engblom’s (2023) doctoral thesis on waiting among refugees, and Helander’s doctoral thesis on DNA testing for family reunification among refugees (Helander, 2023).

In the following sections, the primary objective and research questions of the study are defined, before giving a brief account of the historical context of Swedish immigration policies. Thereafter we explain which methods were used in gathering and analysis of empirical data before delineating the main theoretical concepts and perspectives that have guided our analysis. We then present our analysis in four sections structured around central themes within the dynamics of deservingness and temporal bordering in processes of family reunification. Finally, the main results and insights are presented in a concluding discussion.

## 2 Research question

The primary objective of the article is to investigate deservingness and temporal bordering in Swedish family reunification, exploring how they produce and reproduce global hierarchies of transnational mobility. The main research questions are: *How do deservingness and temporal bordering affect the transnational mobility of families wishing to reunite in Sweden? How do these mechanisms reproduce social hierarchies in transnational mobility?*

## 3 A brief history of Swedish immigration

The Swedish history of migration includes periods of heavy emigration (from 1850 to 1930, about 1.2 million citizens emigrated, mainly to the United States) as well as several periods of extensive immigration, often followed by sudden turns towards more restrictive immigration policies when certain groups have been seen with suspicion or as a “threat” towards Swedish safety or welfare (Byström, 2017; Demker and Malmström, 1999; Lundh and Ohlsson, 1999). Beginning in the mid-1990s, Swedish immigration policy evolved into one of the most liberal in Europe as the country granted a relatively high number of residence permits to asylum seekers (Engblom, 2023) and rarely applied requirements for reunification (Borevi, 2014). In 2015, during the so-called “long summer of migration,” (Mezzadra, 2018; Rozakou, 2020) Sweden was alongside Germany the country in Europe who received the most asylum-seeking refugees. In times when most other European countries were closing their borders for refugees, Sweden’s political leadership continued to defend the relatively open policies (Engblom, 2023).

However, during the fall of 2015, the support for the extensive refugee reception declined, partially due to the authorities’ inability to ensure a humane reception, resulting in a severe shortage of asylum accommodations and an increased workload at the Swedish Migration Agency (hereafter SMA) extending the processing times for asylum applications from four and a half months in 2014 to almost one and a half year by 2018 (Engblom, 2023). Meanwhile, several municipalities complained they did not receive enough resources from the state to manage the situation locally. Politicians on the right side of the political spectrum capitalized on these reports, putting further pressure on the government to implement restrictive measures, prompting a shift in Sweden’s immigration discourse. On November 24, 2015, Stefan Löfven, Swedish Prime Minister and leader of the Social Democratic Party, proclaimed that the country needed a “breathing space” from immigration, and presented a list of restrictive policy changes of which most were implemented in summer 2016 (Engblom, 2023).

In addition to stricter criteria for being granted asylum, a key part of the government’s aim to reduce refugee immigration was tightening family reunification by introducing maintenance requirements, including proof of income and adequate housing (Borevi, 2018). Since then, restrictive policies have intensified, particularly under the current government, consisting of three liberal and conservative right-leaning parties, with active support from the Sweden Democrats, a far-right party known for its strong anti-immigration stance. In a document called the Tidö Agreement, the four parties specify their joint ambition to minimize asylum immigration and increase deportations (Via-TT, 2022). While mainly targeting refugee immigration, these changes have lasting effects also on other groups applying for reunification. In this article, we therefore explore the consequences of these policies on persons with different legal status, including Swedish citizens, work migrants, and refugees.

## 4 Data and methods

The article draws on data from two doctoral projects. The first is based on ethnographic fieldwork in a small industrial municipality in mid-Sweden, between 2017 and 2018. The research opted for a broad understanding of how refugee reception affects rural towns and villages, and how refugees navigate such places. Waiting emerged as a central facet of the experience of refugees and would later become the main theme of the dissertation (Engblom, 2023). In addition to waiting for the processing of asylum cases, waiting for family members was a recurring theme in interviews and conversations. Besides extensive participatory observations and informal conversations, the material included 39 interviews, both in-depth and semi-structured, with refugees in different legal situations. In this study, four of these cases are highlighted, although the entire qualitative material has helped ground the broader analysis.

The second doctoral project focused on waiting within family reunification processes. It employed joint and individual semi-structured interviews with couples or one partner who wanted to or already had reunited. Participants included both persons waiting in Sweden and abroad, mirroring the transnational nature of the process. Open-ended questions regarding the process toward reunification (planning, decision-making, separation, and potential realization of reunification) were asked. Data collection took place between 2021–2023, either online or in person, and encompassed a total of 21 reunification cases. The sponsors had a variety of legal statuses, including work permits, study permits, permanent residency (based on asylum and previous work permit), and Swedish citizenship. In this paper, eight cases are highlighted.

Combining data from two projects comes with several challenges. Data collection and primary analysis were undertaken by two different researchers at different times and with slightly different aims and approaches. In the first project, participants reflected more broadly on both past experiences and their present situation, whereas the second centered around a set of pre-prepared themes focusing explicitly on waiting and family reunification. This made the nature of the data of the two projects slightly different and thus comparisons difficult. However, this article does not pursue a comparative analysis, but uses different cases to illustrate important elements of Swedish family reunification, exemplifying tendencies of broader mobility regimes. For this aim, the study has strongly benefited from the distinct focus of one project on refugee migration and the broader approach of the other.

In both projects, interviews were transcribed verbatim. The two authors then discussed intersections between their material regarding experiences of waiting in family reunification. Codes were created based on the factors identified as most significant in influencing one’s reunification experience. Four themes were then singled out as central for the article (see section 5). The four cases included from the first doctoral project were chosen to illustrate the diverse experiences of people often labelled “irregular,” nuancing stereotypic understandings of forced migration. The eight selected from the second doctoral project were chosen for their ability to illustrate how the recent turn in immigration policies impacts families with diverse backgrounds and legal statuses, moving beyond the more traditional focus on forced migration.

Children were not directly interviewed in either research project, though they were part of the family constellation in five of the highlighted cases. However, ethical considerations precluded direct interviews with the children. Names and other details that can be used to reveal the true identity of the informants have been pseudonymized following established practices.

## 5 Theory

This article explores two central facets of mobility regimes: *deservingness* and *temporal borders*. By mobility regimes we refer to a diverse array of infrastructures, technologies, laws, organizations, markets, and intra-state agreements, which regulate people’s mobilities. These regimes are complex and do not follow a singular rationale. We adhere to a critique of the notion of the postmodern era as characterized solely by increased openness and travel opportunities (see Shamir, 2005; Salazar, 2021; Glick Schiller and Salazar, 2013; Sheller and Urry, 2006; Kesselring, 2013; Dimitriadis and Ambrosini, 2023). While it is true that a privileged few enjoy such freedoms, most of the global population lacks comparable rights and means for transnational mobility. A defining feature of the contemporary global landscape is rather, according to the critique, the increased stratification of rights, abilities and opportunities for movement in the global social hierarchy (Shamir, 2005). In sociologist Ronan Shamir’s view, global mobility governance is predominantly underpinned by a “paradigm of suspicion” that “conflates the perceived threats of crime, immigration, and terrorism,” targeting specific groups, often along racial, ethnic, and national lines (Shamir, 2005).

Scholars have emphasized the interconnectedness of immigration policy and capital markets and interests (Mezzadra and Neilson, 2013; Philipson Isaac, 2024; Maury, 2020; Mulinari, 2024; Öberg and Sager, 2017). One clear example is policies requiring employment for residency. These policies reveal how legal status is not mainly connected to a human rights perspective aiming to protect the most vulnerable, but by whether the labor market can benefit from their skills. Migrants’ rights are thus conditioned by their capacity and perceived willingness to “integrate” into the host country, by adhering to prevailing norms of what constitutes a successful immigrant (Ataç, 2019; Kissová, 2017; Wernesjö, 2020; Fontanari, 2022; Mulinari, 2024; Marchetti, 2020; Van Oers, 2021; Bech et al., 2017). In this paper, we refer to this reasoning as a *logic of deservingness*. Importantly however, parallel with this deservingness logic, researchers argue that the access to the Swedish labor market is both highly racialized and gendered, disfavoring non-whites, non-Europeans, and women (Agerström et al., 2012; Arai and Vilhelmsson, 2001; Bursell, 2014; Carlsson, 2010; Carlsson and Eriksson, 2014; Kofman, 2018; Mulinari, 2024; Wolgast et al., 2018).

The many studies documenting how bureaucracies and legal processes regulate migrants’ time have led some to talk about a *temporal turn* within migration studies (Amrith, 2022; Andersson, 2014a; Lucht, 2016; Maury, 2020; Mulinari, 2024; Fontanari, 2022; Tazzioli, 2018; Yamba, 1995; Philipson Isaac, 2022). These studies point out that time and temporality play a crucial role in migration processes. It has for example been documented how short-term contracts in the global labor market produce a deep sense of living in temporariness among work migrants (Wang, 2020); how migrant trajectories are not linear but characterized by ever-shifting conditions and opportunities, leading to unpredictability and uncertainty (Amrith, 2020); how short-term student visas push non-EU students into low-skilled work (Maury, 2020); and how refugees encounter bureaucratic processes characterized by a combination of deceleration and acceleration (Griffiths, 2014). These mechanisms, which condition the temporal state of various categories of applicants, are referred to as *temporal borders* (Mezzadra and Neilson, 2013).

This article focuses on *waiting* as a central facet of temporal bordering, and something which reproduces social stratification within family reunification. In the literature, waiting is highlighted as a pivotal component of contemporary mobility and border regimes (Engblom, 2023; Khosravi, 2021; Fontanari, 2017; Griffiths, 2014; Andersson, 2014a, 2014b; Lysaker, 2020; Mulinari, 2024). Waiting is embodied in the implementation of hotspots in border areas like Lampedusa, Italy, and Lesvos, Greece, that ostensibly aim at expediting refugee identification but effectively halt refugees’ onward movement, confining them in camps for extended periods (Tazzioli, 2018). In line with critical theories, we view impositions of waiting as manifestations of power (Bourdieu, 2000) that put individuals’ lives “on hold” (Lysaker, 2020) and “defers” people’s future (Andersson, 2014a). Waiting is not merely an outcome of the workings of power but a productive element in the reproduction of border and mobility regimes (Andersson, 2014a; Engblom, 2023; Jacobsen and Karlsen, 2021; Mezzadra and Neilson, 2013; Mulinari, 2024). This is evident in cases where long periods of waiting affect the applicants’ chances to acquire residency or deter others from applying for residency. As a central mechanism of temporal bordering, waiting plays an active role in the reconfiguration of transnational mobility in Swedish family reunification. Here, the Swedish Migration Agency (SMA) is a key actor in the Swedish mobility regime and in the temporal bordering of family reunification, influencing the duration of applicants’ wait through the pace of their caseworkers. But, as will be shown, the increasing paradigm of deservingness also means that waiting for residency is entwined with other forms of waiting, such as to secure a job or a first-hand contract on an apartment, many times leading to further waiting, and increased uncertainty.

This paper rests on the premise that societal structures, such as legal institutions, education systems, and labor markets both produce and reinforce inequality and social stratification. These institutions contribute to a hierarchization of deservingness, impacting families access to mobility and welfare resources. Inspired by sociologist Beverley Skeggs reconceptualization of Bourdieu’s theory on capitals (Bourdieu, 1984), however, we recognize that individuals navigate their positions with the help of various resources at hand. While Bourdieu limits his model to four types of capital (economic, social, cultural, symbolic), Skeggs means that there are other types of resources people make use of in their everyday lives, emphasizing that social hierarchies are processual and context specific (Skeggs, 2004). From this view, social stratification does not revolve around one single axis, such as class, gender, or race, but emerges from a complex interplay of societal structures, categorizations, institutions, relationships, as well as individual aspirations and actions. This multidimensional understanding of power guides the further analysis, enabling a nuanced interpretation of the social stratifications produced and reinforced by contemporary family reunification, and how individuals navigate these.

Throughout the process of completing this paper, we adopted an abductive approach, meaning that our conceptual understanding of family reunification emerged from a close reading and interpretation of empirical observations, both informed by and refining our theoretical preconceptions. Based on these observations the analysis circles around four themes: the role of legal categorization, the increased importance of the labor and housing markets, waiting as part of a global division of power, and circumventions of temporal borders.

## 6 Data analysis

### 6.1 Legal categorizations as temporal borders

Legal categorizations are central to immigration control and involve processes that classify people into different residence permit types, with each legal category granting varying rights and opportunities. In such bureaucratic processes, factors like national background, vulnerability, employability, and age, play important roles. One aspect that significantly has altered Swedish immigration policies since 2016 is the increased use of time-limited permits, termed an “institutionalization of temporality” by migration scholars Öberg and Sager (2017). In their view, this institutionalization stretches across legal statuses, increasing the sense of insecurity in the lives of a wide range of immigrant categories (*ibid.*).

In her study on international students in Finland, Maury (2020) argues that time-limited residence permits represent explicit forms of temporal borders, *inscribed* into law and legal practices. In addition to this, she argues that waiting *arises* as an implicit effect of the bureaucratic practicalities of adjudicating or renewing residence permits, in effect constituting another form of temporal bordering (Maury, 2020). Maury’s distinction is useful in relation to our material, where people categorized into different legal statuses experience a varying degree of both explicit and implicit temporal borders. This is perhaps most evident in relation to migrants who apply for asylum, who cannot apply for reunification until their own application has been processed, and then need to be prepared to renew their permit after either 13 months or three years depending on whether they are categorized as “beneficiaries of subsidiary protection” or “refugees” as formulated in the 1951 Geneva Convention (SFS 2005:716).

The wait for asylum, scholars have emphasized, produces uncertainty both in regard to the outcome and the length of the process, giving rise to deep anxiety and feelings of powerlessness and directionlessness (Bendixsen and Eriksen, 2018; Brekke, 2010; Drangsland K. A., 2020; Griffiths, 2014). Such experiences can be exacerbated for a number of reasons, including separation from family members (Leinonen and Pellander, 2020) or denial of the right to work during the waiting period (in Sweden, asylum seekers are by default excluded from work possibilities, although they can get exempted if they can prove their national identity with legal documentation validated by Swedish authorities).

One person who was both sad and angry about having to wait for an extended period was Haydar, a Kurdish man from northern Syria who fled to northern Iraq with his wife Zanya when the civil war erupted in 2011. In 2015, he left Zanya behind and joined the many refugees heading towards Europe that year. Arriving in Sweden, the Swedish authorities questioned his national identity, making him go through two language screenings to finally establish his origin. After two years and two months, Haydar was granted a residence permit as a “beneficiary of subsidiary protection,” a category that applies to most Syrian refugees. To his great disappointment, the subsidiary permit came with a time-limit of 13 months and—in accordance with a law put in place between 2016-2019—ineligibility to apply for reunification with his wife Zanya. The two had not met since his departure, and Zanya remained in the perilous Kurdish-controlled areas of northern Iraq. Haydar now needed to upgrade his legal category and obtain a permanent residence permit to be allowed to bring his wife to Sweden. To qualify for that, he needed a permanent employment contract with a sufficient salary to cover both accommodation and “normal living expenses,” amounting to about €1,400 per month. For subsequent reunification, he also needed housing and a salary covering not only his expenses but also Zanya’s, adding about €500 per month to the requirements. For Haydar, the realization of the harsh requirements connected to the legal category he had been placed in, marked the beginning of yet another period of uncertain waiting.

The restrictive measures introduced in 2016 included new differentiations between legal statuses that have clearly altered the Swedish mobility regime. The changes disproportionately affect certain categories of migrants like Haydar, who hold permits based on subsidiary protection rather than the 1951 Geneva Convention. Not only did he have to wait for his first residence permit—something which was heavily extended due to the language screenings—but also fulfill the criteria for a permanent permit, extending the wait for reunification indefinitely.

While asylum-seeking refugees are undoubtfully subjected to a wait filled with uncertainty, Mezzadra and Neilson (2013) caution against uncritically reproducing categorical distinctions such as between “skilled” and “unskilled” labor, between “regular” and “irregular” migrants, emphasizing that many trajectories unfold between these social constructs, and how also those who are highly educated experience temporal borders of various kinds. This complexity surrounding legal categorizations is further reiterated by Öberg and Sager (2017), who note that the “institutionalization of temporality,” where legal temporariness has become the norm in immigration policy, has expanded to encompass more immigrant groups than just forced migrants.

One example of this is Masoud, an Iranian man in his early thirties who arrived in Sweden on a work permit. Unlike Haydar, he only had to wait a few weeks to be granted residency in Sweden, as he had found employment for a company certified by the SMA. While in Sweden, he fell in love with Zahra, an Iranian woman whom he met through an online social media platform. The two became a couple, but Zahra was still in Iran. Since the relationship was not established before Masoud’s move, he would need a permanent residence permit before bringing her to Sweden, due to a policy from 2016 (Swedish Migration Agency, 2023e). To qualify for a permanent residence permit, Masoud was required to work in Sweden for four years. After completing the four years, his application for permanent residence permit was expedited in about four months. Thus, although Masoud’s position in a high-paying sector clearly granted him more security compared to Haydar—his permits were granted quickly, he arrived in Sweden by plane, had a high salary that permitted him to rent a nice apartment and take frequent trips to visit his girlfriend—he was nonetheless subjected to temporal bordering, similar to Haydar, manifested in the temporary legal categories that prevented them from reuniting with their partners.

Another important categorization impacting family reunification is age, as the international definition of a child—someone below the age of 18—also determines who is eligible for reunification with their parents and siblings (Swedish Migration Agency, 2023a). Until 2022, children who turned 18 during the application process were disqualified from reunification. Following rulings in the Court of Justice of the European Union, however, the Swedish courts and the SMA changed their practice in family reunification cases, to assess age at application, not at the time of decision (Swedish Migration Agency, 2023d).

In our material, the 18-year limit affected children waiting both in Sweden and abroad, serving as a reminder of the transnational nature of reunification. Abas, who was sixteen when he in 2015 tragically lost contact with his mother and sister when crossing the Afghan-Pakistani border, managed to reach Sweden on his own and apply for asylum as an “unaccompanied minor.” However, the increased waiting times for asylum applications in the mid-2010s, combined with asylum seekers with Afghan background being down-prioritized by the SMA (Engblom, 2023; Rosengren, 2021) resulted in Abas turning 18 before his asylum application was processed. Thus, although he was legally categorized as a child when registering his asylum case, he was considered an adult when the decision was made. The long case processing time significantly declined his chances of receiving asylum (the approval rate for unaccompanied Afghan children was 78% while the approval rate for Afghan adults was 37%) (Engblom, 2023), and Abas’s application was denied. With this denial, Abas’s prospects of reuniting with his family members—if able to locate them—seemed impossible.

Another case illustrating the significance of the legal distinction between child and adult is that of Nadia—a Syrian teenager whose family arrived in Sweden in 2018. The family, including two parents and five children, fled to western Syria when ISIS gained control over Nadia’s hometown in 2015. Jamal and Mohammed, Nadia’s oldest brothers, were sent to Europe, hoping for future family reunification there. Mohammed settled in Germany, while fifteen-year-old Jamal continued to Sweden. During Jamal’s two-and-a-half-year wait for asylum, Nadia turned 18. Their parents Ibrahim and Moniera nonetheless applied for family reunification at the embassy in Lebanon, returned to Syria, and waited for the decision. After six more months, Ibrahim, Moniera, and their two youngest children were granted residency in Sweden—but not Nadia, since she at that point was considered an adult. Ibrahim and Moniera were happy to safely settle in Sweden but devastated that Nadia was not allowed to join them and worried for her safety. Their only option to reunite was to apply under a narrow clause in the Alien’s Act, but they would first need to fulfil the requirements for a permanent residence permit, including a stable employment.

Nadia’s and Abas’s cases highlight how legal categorizations based on age become a temporal border (Maury, 2020). It is here worth recounting Maury’s differentiation between temporal borders that are *inscribed* into law itself—and thus represent an explicit form of temporal bordering—and those that implicitly *arise* from the practicalities of applying and adjudicating applications. Abas’s and Nadia’s waiting were a combination of both, where the age limit marks a definite legal boundary affecting one’s legal opportunities and where the drawn-out case processing times *arose* primarily from the SMA’s inability to meet the increased workload.

The examples of legal categorizations through residence permits and age outlined so far illuminate how access to family reunification is formed. Categorizations and differentiation of rights effectively work as temporal borders by delaying the time until eligibility. Drawn-out bureaucratic processes coincide with the aging of applicants, creating additional challenges for some seeking reunification. For Nadia, the extensive processing time of her brother’s case meant that she was deemed to stay in Syria, separated from her parents and siblings, until she could find alternative ways to flee the country or until Ibrahim or Moniera could establish themselves in Sweden. Nadia, in this way, became both legally and temporally dependent on her family members, the end of her wait being conditioned on her family’s temporary legal status in Sweden. Moreover, Ibrahim, Moniera and the two siblings who had reunited with Jamal depended on Jamal’s permit both temporally and legally, as the time of the family members’ permits cannot exceed that of the sponsor (Directive (EU), 2003). In both Abas’s and Nadia’s cases, the waiting they were subjected to functioned as a double punishment as it both kept them immobile while also decreasing their chances to settle in Sweden, illustrating that waiting is not only an effect of the workings of power, but also a productive element effectively reproducing power asymmetries.

In anthropologist Shahram Khosravi’s words, the time people spend waiting without the ability to use it productively represents time “stolen” from them (Khosravi, 2018). Khosravi argues, from a Marxist perspective, that the way migrants and other groups have their time stolen should be seen in relation to a broader context of capitalism, where “time is associated with success and money” (2018, p. 40; see also Kissová, 2017; Mulinari, 2024). The term “steal” implies that the practice of stealing time from migrants is intentional. In the case of family reunification, it is difficult to determine whether the time families spend waiting is a deliberate mechanism of exploitation. This does not mean, however, that people do not feel as if their time is stolen from them. Neither does it exclude the possibility that capital actors benefit from their waiting, and that the waiting thus plays part of reinforcing structural inequalities, a subject we will turn to in the following section.

### 6.2 Deservingness and the temporal borders of labor and housing markets

In 2016, Sweden joined most other European countries by introducing a number of maintenance requirements for family reunification. These regulations illustrate a departure from a perspective where family reunification is seen as a Human Right (UNHCR, 2024) and a means to strengthening social inclusion, towards a perspective where applicants must deserve reunification by first establishing themselves on the housing and labor markets. We refer to this shift—where financial self-sufficiency has become a prerequisite for societal integration, rather than its ultimate goal—in terms of deservingness (Bech et al., 2017; Kissová, 2017; Van Oers, 2021). A major consequence of these developments is the intensified influence of labor market dynamics on mobility opportunities. This supports Mezzadra and Neilson’s argument that immigration control does not solely function to exclude migrants from national territory, but is part of a broader capitalist structure, with the aim of ensuring a steady supply of labor while also minimizing the risks associated with an unbalanced immigration (Mezzadra and Neilson, 2013). In their view, the different periods of waiting migrants are forced to endure represent “temporal delay[s] that stratify movements into the national labor market and polity” (*ibid.*, p. 150). Building on Mezzadra and Neilson, this section explores the interplay between labor and housing market dynamics, deservingness, and temporal borders.

Maintenance requirements within family reunification apply to all Swedish citizens who wish to bring a family member, unless the citizen is returning from living abroad and has a “well established” relationship with their partner (Swedish Migration Agency, 2023a). A citizen in Sweden wishing to reunite with a partner and two children under the age of six needs to prove housing of sufficient size and standard as well as a disposable income of 16,571SEK (€1,479) after rent has been deducted. The requirements also apply to *beneficiaries of subsidiary protection* or *refugees* in accordance with the 1951 Refugee Convention, for the latter only if they do not apply within three months after they receive their residence permit. Finally, *work permit holders* must first earn a minimum of 28,480 SEK (€2498) before tax to obtain a work permit (before November 1st 2023, the required amount was about half). For reunification, they need to provide proof of sufficient income to cover living and housing costs, calculated similarly to the groups above, but do not have requirements regarding the size and standard of housing (Swedish Migration Agency, 2023c).

The unequal impact of maintenance requirements becomes evident when contrasting the situation of employed and unemployed individuals, clearly demarcating that deservingness is connected to labor market participation. Those who are employed do not need to worry about reaching the requirements, as long as their employer complies with Swedish labor laws and collective agreements. However, this does not mean that employed applicants find the waiting process easy. In our material, several persons who were employed felt stuck in jobs they disliked, as the regulations require an employment with a steady income extending at least one year after the SMA’s decision. And while Öberg and Sager (2017) find the feeling of being stuck as connected to an increased issuing of temporary permits, our findings also suggest that citizens, whose residency is secure, nevertheless experience these emotions since their *partner’s* residency is conditioned on their own performance.

This can be seen in the situation of Irina and Björn, a couple whose case at first glance concurs with the prototype of deserving and hypermobile migrants. Irina is a Russian woman who said she had a “very good career” when she left Russia for Sweden together with her husband Björn, a Swedish citizen, and their children in the spring of 2022. Hoping to be exempted from the requirement to apply from abroad, they decided to travel jointly to Sweden, and wait together. In many ways, the couple was well-off: they owned a house in Sweden, had savings, and Björn earned good money in his current job. While waiting for the application for family reunification to be processed, however, Björn was tied to a job he disliked. Before leaving Russia, he had developed plans to work full time on an art project, but the current immigration regulations kept him from materializing those plans. Irina now feared that Björn’s bitterness of having to put his plans aside would hurt the couple’s relationship. Meanwhile, as a family reunification applicant she was not allowed to work by law in Sweden, causing her great frustration. Irina, used to having a high-status job, felt uneasy because of the dependency on her husband and in “a semi-legal position—having nothing.” Considering that gender equality and women’s autonomy and financial self-sufficiency are heavily embraced in the Nordic context (Kofman, 2018), and often a top priority in many integration projects (Engblom, 2023; Lundstedt, 2005; Lundström, 2018) it is somewhat ironic that incoming wives like Irina are prohibited from working.

Another person who expressed feeling stuck in the labor market was Ali, an IT professional from Pakistan, who had been planning to move to Sweden with his wife and child for a few years before making the transition. He said half-jokingly that while looking for work in Sweden from his home in Pakistan, employers probably thought that he was “just some guy from the far east, probably do not know anything.” After some time, though, he received job offers from several companies and accepted one. He went to Sweden without his family, anticipating they would join him after one or two years. He explained he first wanted to find a home for the family and make sure his employment became permanent before the family’s move, which meant staying at least for a mandatory six-month trial period. He also realized that the family would need time to organize the many papers required by the SMA, including marriage certificate, birth certificates for his wife and son, and passports. In contrast to the case of Björn and Irina, however, Ali’s status as a work permit holder made his residency in Sweden dependent on his employer. Reflecting on his current employment, he admitted he was “being underpaid. Compared to my/… /level of experience and everything.” Despite this, he did not ask for a raise, afraid of losing his position and with that his right to remain in Sweden. In this way, Ali’s situation was similar to that of the student migrants studied by Maury (2020), who felt obligated to accept jobs below their educational level to maintain their residency. Seen from a structuralist viewpoint, the maintenance requirements thus help supplying the labor market with a work force that is kept temporarily immobile (Maury, 2020; see also Öberg and Sager, 2017).

Our material also includes interviews with individuals who sought reunification but were not eligible because of unemployment. Such was the situation of Rachel, an Eritrean woman in her early thirties. She left Eritrea and her baby daughter, Salem, in 2009, and spent five years en route before arriving in Sweden and applying for asylum in 2014. Two years later, she was granted asylum in accordance with the refugee convention and thus received a three-year residence permit. Her hope was that a residence permit in Sweden would allow her to reunite with Salem, her then nine-year-old daughter. As mentioned earlier, Swedish law stipulates that refugees with three-year permits are eligible for family reunification without fulfilling the maintenance requirements if they apply within a three-month period. Unaware of the three-month time-limit, Rachel missed the opportunity to bypass the requirements. She therefore needed a stable job with an income high enough to cover her rent plus the standard living costs for both her and her daughter, in 2016 totaling an equal amount of about €1,800 to €2,300 per month. Apart from the eight years they had already been separated, Rachel and her daughter had to wait until Rachel could meet the requirements to see each other again.

Rachel had applied for several jobs but never received a response. Her difficulties reaching employment are also reflected in statistics on structural inequalities in the labor market. In 2020, Swedish-born persons were almost 25 percentage points above non-European-born residents in employment rate (Swedish Public Health Agency, 2023). Moreover, structural racism in the Swedish labor market has been well-documented (Agerström et al., 2012; Arai and Vilhelmsson, 2001; Bursell, 2014; Carlsson, 2010; Wolgast et al., 2018). As emphasized by Mulinari (2021, 2024), the situation for racialized women such as Rachel seeking employment in Sweden is particularly challenging. A key factor perpetuating this structural inequality is that many women lack the necessary documentation to verify their previous work experiences, leading to a form of “invisibilization” of work (Mulinari, 2024). Additionally, in capitalist societies, deservingness is only connected to certain types of work, excluding unpaid work carried out within the domestic sphere, on family farms, and similar settings. Thus, although Rachel had been an invaluable resource in the Eritrean household where she grew up (taking care of small children, cleaning, cooking) these experiences had little value in the Swedish labor market.

Lacking employment, Rachel attended Swedish for Immigrants (SFI), a course required of newly arrived refugees to receive allowances from the state with the ultimate aim of societal integration preferably through work. But Rachel’s situation became a vicious cycle as her feelings of stress and immense longing for her daughter impacted her mental health negatively, and therefore also her ability to learn Swedish and her chances of getting a job, which in turn affected her ability to live with her daughter. And considering the above discussion on racial discrimination on the labor market, Rachel’s prospects of finding a stable employment in Sweden seemed very limited.

In addition to the financial requirements, the housing requirements place further pressure on applicants. Access to housing is directly linked to the labor market, though, as most landlords require tenants to demonstrate proof of steady income when signing contracts. Additionally, it is necessary to place the housing requirements in relation to an already strained Swedish housing market, to grasp its impact (Grander, 2020). In many Swedish cities, securing rental housing involves being on waiting lists for several years. Rachel, the Eritrean woman, had received a lease contract on a rental apartment through the local municipality administration as part of a broader relocation program. The apartment was too small to qualify for reunification, however, and she therefore had registered on the waiting list for the largest apartment company in the area, where the estimated wait time was about two years. Far from all immigrants are offered relocation in Sweden, however. As demonstrated by Mulinari and Nordling (2022), many people also move between short-term sublet contracts until finding a stable housing solution. These temporary contracts often delay reunification for families, adding another layer to the temporal borders. As an alternative to rental apartments, people can fulfil the housing requirements by purchasing condominium apartments or self-owned houses. Needless to say, this requires financial savings and, in most cases, bank loans which are hard to obtain without steady employment. Ali, the Pakistani IT professional, searched for a long time for a house to rent that would be suitable for his family of three. “I looked for an apartment for one and a half years,” he recounted. “Believe me, I did not get a single viewing. Not a single one!” Instead, he strenuously started saving for the down payment on a house, which took him about a year and a half, whereafter he could finally buy a house and ask his family to register an application for reunification.

In addition to access to employment and financial savings, the time spent in the country also represents an important asset to navigate the housing market. This is evident from the Swedish citizens interviewed of whom only one, Anders, was rejected for not meeting the housing requirement. Anders was rejected because he held a rental contract for a student apartment, which the SMA did not consider a stable enough housing—something he had not realized would impact the application. The rejection prolonged the separation from his partner, who lived in Canada, as they had to wait for an appeal, adding to the year and a half they had already been waiting for a decision. Anders was devastated by the denial and extended delay caused by the appeal process and said it had severely hurt his and his partner’s view on the Swedish migration system. To avoid a second rejection, he had a backup plan: he had two other apartments available for him to move into which would fulfill the requirements. The fact that he had been living in Sweden throughout his life and been registered in apartment queues for many years led to a clear advantage on the housing market in comparison with newly arrived migrants. This in turn increased his opportunities to meet the housing requirements and, consequently, reunite with his partner.

The restrictive shift in reunification policies is part of a broader logic of deservingness where market dynamics increasingly impact mobility opportunities. In the above examples it becomes clear that maintenance requirements make applicants dependent on labor market actors, underlining previous researchers’ findings that employment has become a means for societal inclusion rather than its goal (Bech et al., 2017; Kissová, 2017; Van Oers, 2021). Furthermore, these actors reinforce interdependencies among family members, as the sponsor’s work and housing situation directly affects the chances of family members’ mobilities.

Importantly, in a strained labor market, the thresholds to reach employment are heavily stratified, where some groups face greater challenges than others. In line with Kofman’s comparative research on family reunification in Europe, we find that work experience, level of education, and economic capital are key factors influencing stratified mobility opportunities (see Kofman, 2018). Additionally, our material reveals that gender, race, mental health, and age are also crucial aspects to take into consideration, underscoring the need to apply a multidimensional and intersectional perspective on family reunification.

For many, achieving employment and securing adequate housing takes a significant amount of time, if ever realized. Thus, the restrictive requirements do not only represent an increased connection between labor and housing market dynamics and immigration regulation, but also between deservingness and temporal borders, further delaying the time many families spend waiting in separation.

### 6.3 Unequal conditions of waiting under the global visa regime

Restrictions in mobility regimes often follow divisions between Global North/South and East/West (de Vries and Spijkerboer, 2021). At the same time, as discussed by Kissová (2017), the increased securitization of immigration targets some groups—especially those associated with Islam—as potential hazards against the safety of citizens and national welfare, reinforcing racialized asymmetries (Gardell, 2015; Kissová, 2017; Shamir, 2005).

People targeted under the so-called paradigm of suspicion (Shamir, 2005) often also face restrictions in the global visa regime. In Sweden, citizens of countries associated with refugee flows are often denied visas (see Swedish Migration Agency, 2023b), and EU regulations even provide a list of countries whose citizens must have a visa to enter Schengen territory. The list has been criticized for following old colonial lines, categorizing countries into “positive” (not requiring visa) and “negative” (requiring visa) countries (Maury, 2020). In Mezzadra’s and Neilson’s words, this system reflects a “continental drift” where borders between regional units like the EU, the US, Middle East and East Asia expand (2013, p. 54). Visa regulations impact family members opportunities to meet during extended waiting times. As emphasized in transnational family studies, such visits are key for the endurance of transnational relationships (Baldassar et al., 2007).

In interviews where visas were brought up, both Iranian Masoud and Syrian Haydar said their partners had been denied visas to visit them during the wait. For Zanya, Haydar’s wife, it was because Syria lacked visa agreements with Sweden. For Masoud and Zahra, the closure of the Swedish embassy in Iran during the Covid-19 pandemic made applying impossible, followed by waiting times for embassy appointments of 10 months. When the pandemic restrictions were finally lifted, Zahra‘s visa was, as for many other Iranians, denied. However, in contrast to Haydar, Masoud received a monthly salary high enough to afford meeting Zahra in third countries, such as Turkey, to which both could get visas. This had allowed the two to meet on average once every three months during a three-year-period, apart from when Masoud was waiting for the renewal of his work permit, during which he was not allowed to leave Sweden. Being used to living a mobile lifestyle, Masoud thought of the renewal period as “very annoying.”

Visa restrictions on visiting family members sometimes also jeopardize the right to reunification. According to the caseworkers’ handbook on migration cases, the duration of the relationship and the frequency of meetings between partners are two “objective grounds” used to assess the seriousness of a relationship. The guidelines state that the shorter the relationship, the more frequent the meetings between the couple are needed (Swedish Migration Agency, 2024). These evaluations form part of the bureucratic process of distinguishing what is often phrased as potential “bogus relationships” from couples who truly “deserve” reunification, in the eyes of immigration authorities (Bonjour and de Hart, 2021). As part of the securitization logic mentioned earlier, this deservingness categorization may spill over to case workers’ practices. In her doctoral thesis, Rosén (2010) studied 334 dossiers on reunification from 2002. She found that when assessing the seriousness of a relationship, case workers were less likely to require an interview from sponsors who were men, Swedish citizens, with high education “and a gainful employment,” thus assuming that their relationships were serious. On the contrary, she found that “Muslims and people with Arabic-sounding names are disfavored and are rejected more often” than persons without (Rosén, 2010, p. 283). In temporal terms, those subjected to more doubt and scrutiny also have to wait longer for their reunification cases to be processed.

Jonathan, a Ghanaian citizen, had applied for a Swedish visa without success, and pessimistically proclaimed, during an interview, that “you are denied no matter what,” referring to the skepticism of Swedish authorities to grant visas to Ghanaian nationals. Just as Jonathan had not been allowed into Sweden, his Swedish-citizen partner had not been able to meet him in Ghana either. One reason for this was that she too would need to apply for a visa. As there is no Ghanian embassy in Sweden, she would need to travel 600 kilometers to the embassy in Denmark to apply for a visa. Moreover, she worked full-time and her lack of financial savings hindered her from taking a leave from her job. In addition to this, she was not feeling psychologically well at the time, and travelling abroad was an enterprise she felt she could not manage. In other words, the combination of lack of economic capital, embodied (or psychological) resources (Skeggs, 2004), and Jonathan’s Ghanian citizenship disfavored the two, keeping them separated from each other.

Jonathan worried that the prolonged separation would negatively impact their case. He feared a double punishment, as the inability to meet during the prolonged wait also reduced their chances of having their relationship judged “serious,” disqualifying them from reunification. The procedures thus exemplify another instance where waiting becomes a productive element in the temporal bordering, potentially leading to further waiting (Andersson, 2014b; Engblom, 2023).

For families otherwise relatively benefitting under the “paradigm of suspicion,” restrictive mobility regimes may still prolong the waiting times in separation. As discussed by Bonjour and de Hart, the shock when realizing the obstacles posed by immigration regulation on mixed-status families tends to be especially strong among people with middle-class or upper-class backgrounds who expect that laws and legislation work in their favor, often leading to strong resentments towards authorities (Bonjour and de Hart, 2021). Our data provides several examples of this, among others British-Swedish couple Kurt and Siri.

The couple had few worries as they were living relatively close to each other, and no visa regulations hindered them from visiting each other during the reunification process. Like other British citizens, though, the couple was affected by the 2019 Brexit withdrawal agreement and, in effect, Kurt’s desire to move to Siri in Sweden was seriously hampered. Kurt said:

… the second that the transition period ended and the withdrawal agreement was signed and everything, we started reading about the immigration processes more, and it became more and more clear that actually we’d need to spend, we probably need to spend a lot of time apart, whilst I waited for the visa.

As a result of the Brexit agreement, a new temporal border was erected between people like Kurt and Siri. Although their right to visit each other remained, Kurt no longer experienced the same level of freedom of movement which he previously enjoyed. Had the couple reunited in Sweden a year earlier, when Kurt was still an EU citizen, he could have moved to Siri straight away, without the need to submit an application. However, with the shifting borders of the mobility regime, their forced separation while waiting for their case to be processed became a significant source of frustration.

Almost unanimously, interviewees who were citizens of countries with visa agreements with Sweden complained about the unnecessary money spent on travels to visit their partners—a physically mobile lifestyle they would rather leave behind. Instead, most expressed a clear longing for a settled, more immobile, life. Although these people were highly mobile in physical terms, they felt stuck both socially and existentially (Hage, 2009; Salazar, 2021).

The differential impact of visa regulations, rooted in global hierarchies of mobility and ideas of “the other” (Shamir, 2005), becomes evident in the cases above. For some, like Jonathan and Haydar, the global visa regime becomes an almost insurmountable barrier. For others, like Kurt and Siri, visa-free agreements and financial capital meant they could facilitate meetings during the wait, although it weighed heavy on their relationship and personal lives. For EU-citizens, however, travel is not restricted by any visa regulations and they can relatively freely relocate themselves across national borders. All in all, the current visa regime contributes to making entire populations either “deserving” or “undeserving” of mobility opportunities, perpetuating global hierarchies while prolonging the time families remain separated.

As argued by Mezzadra and Neilson (2013), however, any border (national, regional, local, social, cultural) tends to give birth to a multitude of actions, interactions, and relations, in the ambitions to navigate, or even exploit, the border. In other words, borders are not merely repressive, but also productive (*ibid.*). In the next section we show how families deploy strategies and utilize financial or social capitals to creatively navigate the increasingly restrictive mobility regime, thereby re-making transnational geographies.

### 6.4 Circumventing temporal borders and re-appropriating time

Waiting as an experience is often considered synonymous with passivity and immobility. Especially in relation to forced migration, scholars have stressed the negative effects waiting has on individual’s wellbeing (Brekke, 2010; Griffiths, 2014; Ramsay, 2017; Rozakou, 2020). As brought forward by anthropologist Ramsay (2017), however, focusing on the negative effects of waiting risks unwittingly reproducing a stereotypical view of refugees as fundamentally passive subjects, embodying an “temporal otherness” (Ramsay, 2017, p. 204). As an alternative, she urges scholars to account for how displacement and waiting are not limited to refugees, but are increasingly existential aspects for many people caught in precarity and vulnerability in a globalizing context. Comparing the reunification experiences in our material, we see several concurrences between people categorized as “refugees” and other applicants.

Another way to avoid reproducing those in waiting as passive is to account for how waiting is resisted, and how people exert agency under waiting, as argued by Fontanari (2017; see also Bendixsen and Eriksen, 2018; Griffiths, 2014; Rotter, 2016). In her study on refugees living in reception centers in the UK, Fontanari highlights how individuals “carve out” everyday moments for relaxation and socialization, described by her as “practices of time re-appropriation” (2017, p. 45). While her perspective focuses on individual strategies to mentally cope with their situation, we find it important to complement such accounts with a family perspective, as proposed by Bonjour and de Hart (2021), to develop an understanding of how families respond to, navigate, and circumvent legal and temporal barriers (see also Drangsland K. A., 2020).

One couple who tried to find ways to navigate the geographical and temporal borders they were subjected to was Casey and Kim. The two had met in the US where they became a couple, but eventually decided that Casey, a US citizen, would settle with Kim in Sweden. Kim, a Swedish citizen, relied on sickness benefits (*sjukersättning*) due to a disability, a circumstance which they suspected could make their case processing more complicated. The couple was nervous of the outcome of their application, as they knew the benefits were not large enough to meet the financial requirements unless they could get an exemption due to Kim’s disability. The disability also made Kim very hesitant to move to the US, their plan b, since they would lose the sickness benefits if moving abroad.

Restricted by Schengen visa regulations, Casey was only allowed to stay up to 90 days within a 180-day period in Schengen territory, representing a very clear form of temporal border. This arguably positioned them as relatively privileged compared to persons from from countries for which visas are rarely issued, since they were able to stay with Kim for three months at a time. In order to make use of the regulations as much as they could and avoid travelling back to the US in the three-month periods they could not be in Sweden, Casey developed an alternative mobility strategy:

So what I've ended up doing is, on the months that I can't be here [in Sweden], I've gone to countries that are closer than the US is, but I still have to be out of the Schengen area so. I've just ended up in these random countries suddenly living there and dealing with that… it's just like existing in limbo kind of, just having to try to go on with my life, even though everything is very uncertain and strange. It also keeps me from being able to… really make new friends in Sweden, so I'm not really able to interact with a lot of people in person. Or get new connections or anything. Which is…just adding to the exhaustion.

Casey was undoubtedly active while waiting, and they considered the opportunity to discover new cultures interesting. At the same time, the hypermobility and inability to settle was emotionally and economically draining: because of the long stays in Sweden, Casey was unable to keep a stable job in the US and instead financed their living through savings and support from their parents. In light of this, Casey and Kim’s everyday life was penetrated by restrictions on mobility, yet their strategy to circumvent Schengen regulations can be seen as a way to defy visa regulations, thereby challenging current mobility regimes and their temporal borders. Their example further reiterates the necessity to abstain from a dichotomy between immobility as negative, and mobility as positive.

For families facing greater restrictions, such as Haydar and Zanya, circumventing the global mobility regime usually requires financial funds and networks for smuggling. After the couple discovered that Haydar would need a permanent residence permit to reunite, they initiated a plan that included a significant detour: to bring Zanya to Scotland where they had relatives who could provide accommodation, and where her chances of obtaining a residence permit seemed greater than in Sweden. Yet European borders had tightened significantly after 2015, and the EU-Turkey deal in March 2016 closed the Mediterranean route from Turkey to Greece, increasing the risk of Zanya being sent back if detected by Greek authorities (Heck and Hess, 2017). Air travel was the optimal choice, but it required a passport and flight tickets. Without disclosing further details, Haydar mentioned that the project to get Zanya to Scotland involved numerous calls to brokers, and extensive personal loans from relatives, illustrating how access to social capital sometimes can be used to access financial capital, with the final goal of reuniting. Although not formally recognized as legitimate, the family’s ability to access finances and networks of brokers helped the two circumvent the legal, geographical and temporal borders of the global mobility regime.

Zanya and Haydar met for the first time in three and a half years in 2018. The wait did not end there, however: Zanya had made it to Scotland but was not allowed to leave while her case was being processed. On his end, Haydar had to attend SFI in Sweden to qualify for subsidies, so the two had to live separately until the Scottish authorities investigated Zanya’s asylum application. If approved, they hoped to live together, in either Scotland or Sweden.

In Fontanari’s terminology, the way individuals navigate and circumvent the legal, geographical, and temporal barriers separating them, represents a form of “time re-appropriation” (Fontanari, 2017). Our understanding of Fontanari’s concept resonates with Dwyer’s (2009) idea of activity in waiting: it does not refer to a general capacity to act—in this case a *de facto* possibility to change one’s legal situation—but as the capacity to find family time, including shortening the wait in separation, within these systemic restrictions. While we agree with Fontanari of the importance to account for how individuals cope with periods of waiting marked by uncertainty, and avoid portraying them as passive, there is also a risk that the concept “re-appropriation” misleadingly implies that individuals can reclaim or take back the time that is stolen from them, to use Khosravi’s wording. The time and resources people invest in activities to maintain intimacy or shorten the wait until reunification are on the one hand highly meaningful for them. On the other hand, these activities are imposed, directly or indirectly, due to regulations surrounding reunification and therefore represent time wasted (Mulinari, 2024).

## 7 Discussion

This paper set out to explore how *deservingness* and *temporal borders* in Swedish family reunification produce and reproduce hierarchies of transnational mobility. Drawing on qualitative interviews with families from a wide array of backgrounds the article expands existing research on temporal borders, which has largely focused on forced migrants, by demonstrating the broader impact of restrictive reunification policies in Sweden.

A key element of the new policies, introduced in 2016, is the financial and housing requirements. As discussed in previous literature, such requirements reflect a broader trend of deservingness, favoring those who can demonstrate financial self-sufficiency through employment (Bech et al., 2017; Kissová, 2017; Van Oers, 2021; Öberg and Sager, 2017). We contribute to this literature by applying a multidimensional perspective on power, analyzing how intersections of financial status, educational level, nationality, age, legal categories, gender, and bodily and mental abilities differentially shape mobility opportunities. Our data illuminates how the increasing connection between immigration policy and labor and housing markets reproduces existing power disparities, where each sector tends to favor able-bodied, white, educated and financially resourceful people from the global North/West, making them more likely to qualify for—i.e. be deserving of—family reunification. Crucially, our findings reveal that the maintenance requirements function as a temporal border, prolonging the time families must wait in separation, particularly when the sponsor faces difficulties in securing stable employment and housing in Sweden. Additionally, long case processing times and the introduction of time-limited residence permits further cement an “institutionalization of temporality” (Öberg and Sager, 2017) within the Swedish mobility regime. In light of this, we argue that the interplay between temporal borders and deservingness represents a pivotal dimension in the reproduction of social stratifications within family reunification.

Our research moves beyond a narrow focus on *individuals* within migration studies—often referred to as methodological individualism (Povrzanović Frykman, 2018; Udehn, 2002)—and adopts a *family perspective* to understand the impact of temporal borders and deservingness. While previous research has shown how restrictive immigration policies foster dependencies on landlords and employers (Maury, 2020; Mulinari and Nordling, 2022; Öberg and Sager, 2017), we emphasize that these dependencies also extend to family members. This is evident in situations where a sponsor’s temporary permit determines the length of time the family is allowed to remain in Sweden. Furthermore, the sponsor’s ability to demonstrate deservingness has direct implications for the family in terms of mobility, temporal horizons, and rights. In combination with intersections of social factors, such *legal* and *temporal dependencies* shape the family’s mobility opportunities and, consequently, their ability to plan for the future. For instance, a work permit holder might experience racial discrimination and legal insecurity in the labor and housing markets, delaying the family’s visa processes; a Swedish citizen, whose qualifications are officially acknowledged, might struggle to meet the financial requirements due to their reliance on sickness benefits; and a Ghanaian citizen and his Swedish citizen partner, despite meeting all requirements, may still face significant barriers within the racialized global visa regime. These global divisions of power hamper the possibilities of many families to visit each other during the wait. In turn, extended separation might hurt the relationship, but also the evaluation of pending cases, since the frequency of partners’ meetings is considered during the adjudication. In this context, waiting is not merely an *effect* of a restrictive immigration, but also a *productive element* within the stratification of mobility.

The increasingly restrictive immigration policies in Sweden suppress one of the few legal routes that families have towards settlement in Sweden and bring people into extended periods of waiting in uncertainty. For some families, the maintenance requirements make sponsors stuck in jobs they dislike. For others, fulfilling the maintenance requirements becomes an almost insurmountable barrier extending separation for indefinite periods of time. For those whose family members are stuck in war zones, moreover, attaining stable employment and housing becomes a matter of life or death, as the future of their loved ones is dependent on their success in Swedish society. An experience common to all families interviewed in this study, however, was the sense of lacking power over one’s time, causing significant stress, frustration, longing, and often seriously hurting transnational relationships. However, as seen in this paper, even in the most challenging situations people attempt—sometimes successfully—to navigate their circumstances by using different types of resources at hand.

Ultimately, as policies move away from a human rights perspective towards one of deservingness, mobility regimes increasingly dictate who is deserving of being with their family and planning a future with them.

## Data Availability

The datasets presented in this article are not readily available because data contain highly sensitive details on living human individuals. Requests to access the datasets should be directed to rikard.engblom@oru.se; hilda.gustafsson@mau.se.
